# Editorial: How do extreme environments influence psychological functioning for performance?

**DOI:** 10.3389/fpsyg.2022.1094998

**Published:** 2023-01-30

**Authors:** Guillaume R. Coudevylle, Nadia Gaoua, Toby Mündel, Stephen S. Cheung

**Affiliations:** ^1^Laboratory ACTES (EA 3596), University of the French Antilles, Pointe-à-Pitre, France; ^2^London South Bank University, London, United Kingdom; ^3^School of Sport Exercise and Nutrition, Massey University, Palmerston North, New Zealand; ^4^Department of Kinesiology, Brock University, St. Catharines, ON, Canada

**Keywords:** heat, tropical climate, motivation, personality traits, sport

Whether physical or psychological, the individual determinants of work or athletic performance are the subject of a large body of literature aimed at better understanding what makes an individual perform and how their work can be made safer and more productive (Van Yperen et al., 2014; Gomez-Ruano et al., 2020; Chen et al., 2022). For example, given global warming, thermal stress will exact greater strain on both athletes and also emergency crews during extreme weather events, forcing the body to adapt both physiologically and psychologically. Research on the physical and psychological preparation of athletes in a heat context consists of producing knowledge to help athletes cope with thermal constraints but can also be approached as training programs among others (training at altitude or in a hot and humid environment). Similarly, a better understanding of thermal impact on workers such as wildland firefighters can lead to innovations in scheduling, nutrition, hydration, and equipment.

Stress is how the human body adapts when, repeatedly exposed to exercise and/or environmental conditions, it exceeds its initial capacities and adapts. However, when they are not controlled, these extreme environmental constraints, such as high temperatures sometimes coupled with high hygrometry, amplify the stress imposed on the body and expose individuals to significant decrements in performance and increased health risks. A better understanding and management of environmental constraints is therefore a major challenge for athletes, workers, and their support crew. While the physiological responses to extreme environments have benefited from a large body of literature, their influence on psychological functioning is less understood. In this Research Topic, four articles focus on the psychological perceptions, cognitive performance and athletic performance of individuals subjected to harsh environments (tropical conditions, hypoxia, passive hyperthermia). While a first study measured the impact of a constraining thermal condition (i.e., passive hyperthermia) on the cognitive functioning of individuals, the other three studies focused on possible interventions (self-selected motivational music, per-cooling, motivational strategies) to help individuals perform under environmental stress conditions (hypoxia and/or tropical conditions).

Specifically, in the first study, Schultz Martins et al. investigated the role of skin and core temperature and partial pressure of end-tidal carbon dioxide on cognitive function in a series of domains. Indeed, while increased body temperature due to heat stress generally impairs cognitive function, the relative contribution of changes in skin temperature to core temperature requires more clarity. The different manipulations performed on the 11 participants lead the authors to conclude that neither skin temperature nor the maintenance partial pressure of end-tidal carbon dioxide significantly impaired cognitive function during passive hyperthermia.

In the other three studies, authors focused on possible interventions to help individuals perform under environmentally-stressful conditions. The first of these studies focused on music as a tool for mitigating the performance decrements observed in hypoxia. People exposed to hypoxia may suffer from physiological and psychological consequences. At the same time, it is known that music has ergogenic effects to improve psychological factors such as mood, emotion and cognition. For this reason, O'Keeffe et al. asked 13 men to participate in a study designed to test whether self-selected motivational music could buffer the performance decrements observed in hypoxia. To do this, the authors tested the effects of subjective, physiological and physical performance measures in four experimental trials crossing self-selected motivational music (vs. no music) and normobaric hypoxia (vs. normoxia). Their results show positive effects in several of the measures tested indicating that preferential motivational music plays a beneficial role in mitigating the negative impact of hypoxic conditions.

In the second study on possible interventions to reduce the negative impact of environmental conditions, Riera et al. tested the effect of face cooling with cold water (vs. face cooling with neutral water) during high-intensity swimming training on both the core temperature and thermal perceptions in internationally ranked long-distance swimmers during 2 randomized swimming sessions. Among the results, thermal comfort was higher with cold cooling than neutral cooling and thermal sensation was lower with cold cooling compared with neutral cooling. In general, the authors suggest that brain integration of signals from physiological and psychological sources is involved in the changes obtained by per-cooling.

Finally, Coudevylle et al. conducted a mini-review in which they refer to how the tropical climate impacts motivational factors during aerobic performance and the strategies to maintain the motivation of athletes in such conditions. For this purpose, they proposed a theoretical model integrating several theoretical frameworks that need to be tested by empirical studies. The authors nevertheless remind us of the importance of being cautious because under the cover of an increase in motivation and performance, the risk is to push athletes beyond their physiological limits with all the potentially serious consequences on their health.

Although these four studies focused on students and athletes, the ultimate goal is also to investigate other populations whose professional activities are subject to particularly difficult environmental conditions (e.g., gravity, stress, high risk taking, heat). Thus, other high-level athletes such as GP motorcycle or Formula 1 drivers, but also construction workers, firefighters, police and army special intervention forces, and fighter pilots would need to be investigated in order to help optimize their performance and health in different environments.

## Author contributions

GC is responsible for the production of this special issue, including this editorial. GC proposed a first version of this editorial to NG, TM, and SC. Each co-author then amended the document on both substantive and editorial issues. The GC then compiled all comments and changes and inserted them into the final submitted version. All authors contributed to the article and approved the submitted version.
